# The Emerging Role of the Thrombin Receptor (PAR-1) in Melanoma Metastasis - a Possible Therapeutic Target

**DOI:** 10.18632/oncotarget.211

**Published:** 2011-01-25

**Authors:** Gabriel J. Villares, Maya Zigler, Menashe Bar-Eli

**Affiliations:** The University of Texas, MD Anderson Cancer Center, 1515 Holcombe Blvd, Unit 173 Houston, TX

**Keywords:** cancer, melanoma, thrombin receptor, PAR-1, target, oncotarget

## Abstract

Melanoma remains as the deadliest form of skin cancer with limited and inefficient treatment options available for patients with metastatic disease. Within the last decade, the thrombin receptor, Protease Activated Receptor-1, has been described as an essential gene involved in the progression of human melanoma. PAR-1 is known to activate adhesive, invasive and angiogenic factors to promote melanoma metastasis. It is overexpressed not only in metastatic melanoma cell lines but is also highly expressed in metastatic lesions as compared to primary nevi and normal skin. Recently, PAR-1 has been described to regulate the gap junction protein Connexin 43 and the tumor suppressor gene Maspin to promote the metastatic melanoma phenotype. Herein, we review the role of PAR-1 in the progression of melanoma as well as utilizing PAR-1-regulated genes as potential therapeutic targets for melanoma treatment.

## MELANOMA OF THE SKIN

In the United States, more than 68,000 new cases of melanoma were estimated to be diagnosed in 2010, continuing the trend of increased melanoma incidence seen over the past 40 years [1]. Melanoma is the fifth most commonly diagnosed type of cancer in men and seventh most common in women [1]. The lifetime risk of an American developing invasive melanoma is 1 in 55 as compared to 1 in 1,500 in the 1930s [2, 3]. More than 80% of melanomas are diagnosed at an early clinical stage (before regional metastasis) with more than 95% 5-year survival rates for patient's with localized melanoma [2, 4]. However, when melanoma has spread to regional lymph nodes or metastasized to other organs, there is a significant decrease in survival. The 5-year survival rate for melanoma patients with distant metastasis is significantly decreased to 15% [2]. Therefore, it is imperative to determine the molecular events that lead to melanoma metastasis in search for possible therapeutic molecular targets to curtail the disease.

Early stage melanoma is easily treatable and highly curable with surgical resection [5]. However, once melanoma has metastasized, there are limited and ineffective treatment options available for patients [5, 6]. Decarbazine (DTIC) is the only FDA-approved chemotherapeutic agent for the treatment of metastatic melanoma despite response rates of only 15-20% [5, 6]. Furthermore, the duration of the response to DTIC is not sustained, often lasting as brief as 5 months, with only 5% of patients showing a complete response [7, 8]. Clinical studies utilizing adjuvant immunotherapies with interferon α for high-risk stages II and III melanoma, as well as treatment with high-dose interleukin (IL)-2 for stage IV melanoma, showed limited response rates of less than 15% [9-12]. Identification of melanoma oncogenes, tumor suppressor genes and information gathered from microarrays have advanced our understanding of the molecular mechanisms of melanoma [6]. Further studies in these areas will allow for a clearer understanding of the process of melanoma progression that might lead to the development of urgently needed clinical therapies for metastatic melanoma.

Our laboratory has been studying novel targets involved in the progression of melanoma to develop better therapeutic targets for metastatic melanoma. One of these genes that plays a key role in the progression from non-metastatic to metastatic melanoma is the thrombin receptor, Protease Activated Receptor-1 (PAR-1).

## PAR-1

PAR-1 has been found to be involved in the progression of several cancers including breast [13, 14], colon [15, 16] prostate [17, 18] and melanoma [19-22]. As such, PAR-1 has significant roles not only in coagulation, wound healing and inflammation, but also in the progression of several cancer types. PAR-1 was first identified by Vu et al. in 1991 [23] and remains the most studied and best characterized protease-activated receptor.

**Figure 1:**
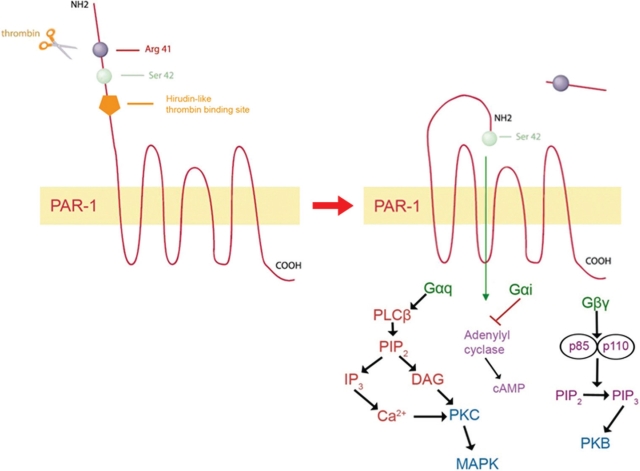
Activation and signaling of PAR-1 Thrombin, the most potent activator of PAR-1, binds to the hirudin-like binding domain on the N-terminus of the receptor and proteolytically cleaves between Arg 41 and Ser 42 in an irreversible manner. Ser 42 now acts as a tethered ligand to activate PAR-1. PAR-1 signals through G-protein subtypes, Gα_q_, Gα_i_, and Gβγ to activate PKC and MAPK, inhibit adenylyl cyclase as well as activate PI3-K and PKB.

The thrombin receptor belongs to a family of seven transmembrane G-protein-coupled receptors with a unique method of activation. Unlike typical ligand-binding interactions that occur with other types of G-protein receptors, PAR-1 activation occurs through proteolytic cleavage of the N-terminal domain of the receptor by serine proteases. Although thrombin is the most effective and potent activator of PAR-1, activation can occur through other ligands, such as coagulation factor Xa, trypsin, granzyme A and plasmin [24, 25]. PAR-1 can also be proteolytically cleaved and activated by matrix metalloprotease-1 (MMP-1) in breast cancer cells [26]. PAR-1 activation by thrombin involves the anion-binding exosite of thrombin binding to the PAR-1 amino terminus in an acidic region spanning amino acids 51 to 63 [23]. This highly acidic region, termed the hirudin-like binding site, is similar to the amino acid sequence of the leech anticoagulant peptide, hirudin [27]. This region increases the affinity and potency of thrombin for PAR-1 [27]. PAR-1 also contains a thrombin cleavage site between Arginine at position 41 and Serine at position 42 in the PAR-1 sequence. Thrombin proteolytically cleaves the PAR-1 N-terminus in an irreversible manner, thereby forming a new amino terminus with Serine 42 acting as a tethered ligand to activate the receptor [23, 28] (Figure 1)

Once activated, PAR-1 signals through the activated G-protein subtypes, Gαq, Gαi, Gα12,13, and Gβγ resulting in downstream activation of several signal transduction pathways, such as the phosphoinositide-3 (PI-3) kinase, the mitogen-activated protein (MAPK) kinase, Rho kinases, as well as increases intracellular Ca^2+^ and activation of protein kinase C (PKC) [29] (Figure 1). PAR-1 is primarily involved in mediating the effects of thrombin, which is recognized as a potent mitogen in cancer and tumor metastasis through sustained ERK 1/2 activation [30]. Protease-Activated Receptors can also signal in response to proteases that stem from the tumor and the tumor microenvironments such as MMP-1 [26]. Activation of PAR-1 by thrombin also results in overexpression and secretion of pro-angiogenic and invasive factors such as IL-8, VEGF, PDGF2 and MMP-2 [31].

## PAR-1 IN MELANOMA

Thrombin generation occurs through multiple pathways including the activation of coagulation factors often seen in several cancer types, thereby leading to PAR-1 signaling [31]. In melanoma, as well as in other cancers, tissue factor (TF), an initiator of the coagulation cascade, has been found to be upregulated [32]. This results in expression of thrombin by activation of the coagulation factor X through expression of tissue factor [32]. Furthermore, several studies have also demonstrated that TF is constitutively expressed in melanoma cells and can activate thrombin in a coagulation-independent manner, thereby promoting melanoma metastasis through PAR-1 activation [33, 34]. Moreover, thrombin activation results in the cleavage of fibrinogen into fibrin. These fibrin deposits are located in the tumor microenvironment and store active thrombin that is released upon degradation of fibrin by plasmin [31, 35, 36].

In an experimental murine lung metastasis model of melanoma, B16F10 murine melanoma cells transfected with the PAR-1 gene resulted in a 40-fold increase in pulmonary metastasis, thus demonstrating that PAR-1 was the rate-limiting factor in thrombin-enhanced pulmonary metastasis [37]. Conversely, when B16F10 cells were treated with a specific thrombin inhibitor, cell migration was suppressed and in vivo metastasis was decreased [38].

PAR-1 has also been found to be overexpressed in human metastatic melanoma cell lines as compared to non-metastatic cell lines [21]. Furthermore overexpression of PAR-1 is predominantly seen in malignant melanoma tumor specimens and in metastatic lesions as compared to benign nevi and normal skin by immunohistochemistry [20]. Utilizing an automated quantification laser scanning cytometer on a tissue microarray platform, our laboratory has also found a significantly higher percentage of PAR-1 positive cells in metastatic melanoma as compared to both dysplastic nevi and primary melanomas [22].

## TRANSCRIPTIONAL REGULATION OF PAR-1 IN MELANOMA

The transition from the radial growth phase to the vertical growth phase in the progression of human melanoma is associated with the loss of the transcription factor Activator Protein-2α [39-41]. Loss of AP-2α correlates with a concomitant increases in PAR-1 expression [21].

Analysis of the PAR-1 promoter region reveals multiple AP-2α and SP-1 binding sites as well as two overlapping SP-1 and AP-2α transcription factor binding motifs within the proximal 3' region, thereby suggesting that these transcription factors mediate PAR-1 promoter activity [42]. Utilizing ChIP analysis, Tellez et al. demonstrated that AP-2α is bound to the PAR-1 promoter in low and non-metastatic melanoma cell lines while SP-1 is bound to PAR-1 in metastatic melanoma cell lines [21]. Furthermore, it was seen that AP-2α and SP-1 bind in a mutually exclusive manner in one of the overlapping binding regions [21]. Thus, in melanoma, the low levels of AP-2α enable SP-1 to bind to its motif in the PAR-1 promoter, which results in PAR-1 gene activation. Non-metastatic melanoma cells express AP-2α. In these cells, the PAR-1 promoter is bound by the AP-2α transcription factor thereby suppressing PAR-1 gene expression [21].

## IN VIVO EFFECTS OF SILENCING PAR-1

Recently, utilizing lentiviral based shRNA, we stably silenced PAR-1 by more than 80% in two human metastatic melanoma cell lines, A375SM and C8161 (high expressors of PAR-1), and injected these cells subcutaneously (to assess tumor growth) and intravenously (to assess experimental lung metastasis formation). We found significant decreases in tumor growth of PAR-1-silenced melanoma cells as compared to cells transduced with a non-targeting (NT) control shRNA. Moreover, significant inhibition of experimental lung metastasis formation was detected in PAR-1-silenced cells [43].

Although significant differences were seen in vivo, the use of a lentiviral delivery system as a therapeutic modality for the delivery of shRNA in melanoma or other cancers brings about several problems including the possibility of viral integration into an area where off-target gene expression can be affected thereby leading to the development of diseases or cancer. Furthermore, the shuttling system that moves the shRNA from the nucleus into the cytoplasm for processing and gene silencing has the potential to be overwhelmed, such that exportin-5, involved in shRNA and miRNA shuttling, might be overloaded and fail to export essential miRNAs or other factors needed for normal cell functioning [44]. Moreover, for shRNA to be processed, Dicer must be present in cells. Merritt et al. found that Dicer expression was variable among ovarian cancer specimens and further demonstrate that low-Dicer expressing cells failed to significantly silence target genes when utilizing shRNA [45]. For effective use of lentiviral based shRNA, the consistent expression of Dicer must be present for gene silencing to occur.

Thus, the use of viruses as a delivery tool for clinical therapies has several adverse effects including toxicities, overwhelming nuclear export mechanisms, as well as unwanted and unpredictable genetic alterations after viral integration [46, 47]. In recent years, much research has gone into finding not only effective but also safer alternative technologies to deliver siRNA to tumors including nano-liposomes [47, 48].

Liposomes are lipid vesicles that allow for the entrapment of not only siRNA but also various types of drugs or small molecules. By incorporating siRNAs into liposomes, they are protected from degradation and thus, the half-life and potency of the siRNA is increased [49]. Within the last 5 years, the use of siRNA packaged into neutral 1,2 dioleoyl-sn-glycero-3-phosphatidylcholine (DOPC) liposomes have been utilized effectively in viv*o* against EphA2 and FAK to treat ovarian cancer [50, 51] as well as neuropilin 2 for the treatment of colorectal carcinoma [52]. These studies demonstrated effective silencing of target genes with limited toxicities utilizing low-dose siRNA-DOPC delivered via intraperitoneal injections [48, 50]. The liposomes were found to rapidly enter the liver, spleen, kidney and reticuloendothelial system (RES) along with the target tissue [47]. Nevertheless, the target genes were silenced in vivo, demonstrating the effectiveness of this delivery system as a potential therapeutic modality in cancer and other diseases.

Although we showed that metastatic melanoma cell lines transduced with lentiviral-based PAR-1 shRNA lost their potential in forming melanoma tumors and metastatic lung colonies, the goal of most melanoma research is now aimed at developing therapies from the benchside to the clinic. Because of this, we wanted to use siRNA-DOPC nano-liposomes as an alternative delivery system that is safer and more feasible, yet effective.

Similar to the results obtained using lentiviral based silencing of PAR-1, we saw a significant decrease in melanoma tumor growth and experimental lung metastasis in melanoma tumor-bearing mice treated with PAR siRNA-DOPC as compared to NT siRNA-DOPC-treated mice [43]. Moreover, Tumors from PAR-1 siRNA-DOPC and NT siRNA-DOPC-treated mice were analyzed for invasive and angiogenic factors including MMP-2, IL-8 and VEGF. Immunohistochemical analysis revealed a decrease in these angiogenic and invasive factors after PAR-1 silencing. Furthermore, through CD31 staining, we observed a decrease in the number of blood vessels in PAR-1-silenced tumors as compared to NT siRNA-DOPC treated mice. In contrast, blood vessels were largely dilated in NT siRNA-DOPC tumors.

It is important to note that the PAR-1 siRNA entrapped in the DOPC liposomes is not targeted specifically to the tumor cells. Our experiments however, utilized a PAR-1 sequence specific for human PAR-1 that would not recognize murine PAR-1. Furthermore, PAR-1 is not expressed on mouse platelets and plays no role in platelet aggregation. Although this was beneficial for our experimental design, it did not allow us to determine possible side effects of systemic delivery of PAR-1 siRNA in terms of platelet aggregation or coagulation. Nevertheless, the use of PAR-1 siRNA might still offer a plausible therapeutic target as PAR-1 does not interfere with thrombin generating fibrin (which subsequently forms a clot) nor does it interfere with other mechanisms that activate platelets, such as coming into contact with collagen from damaged blood vessels [53]. Furthermore, platelets can be activated by PAR-4 at higher concentrations of thrombin [29]. Therefore, a decrease in PAR-1 might not have such severe effects on coagulation as compared with other antithrombotic agents [53]. This could be corroborated by silencing the human PAR-1 equivalent in mice, PAR-3, with systemically delivered siRNA-DOPC in vivo.

Nevertheless, we sought to elucidate novel downstream target genes of PAR-1 that might not only increase our understanding of the mechanisms by which PAR-1 affects melanoma growth and metastasis, but might also be targeted by siRNA-DOPC therapies.

## REGULATION OF CONNEXIN 43 BY PAR-1

To identify downstream target genes of PAR-1 that might contribute to the metastatic phenotype of melanoma, our laboratory performed cDNA microarray studies. This strategy led us to identify Connexin 43 (Cx-43) as a target gene of PAR-1 [54].

Intracellular membrane channels called connexins not only allow for the passage of molecules less than 1.2 kD to pass between adjacent cells but have also been described to function as membrane proteins with adhesive properties [55, 56]. The attachment of tumor cells in transition from a primary site to a secondary organ site requires the attachment as well as the migration of tumor cells through the vascular endothelium. This communication between tumor cells and endothelial cells has been shown to be mediated by connexins and are critical to tumor cell extravasation at the metastatic site [57-59].

Increased Cx-43 expression has been observed in several cancers, including breast cancer [60] and gliomas [55]. In fact, decreased Cx-43 expression reduced adhesion of breast cancer cells to the pulmonary endothelium. [61]. In melanoma, increased coupling of Cx-43-expressing murine melanoma cells to vascular endothelial cells was previously reported [57]. However, the mechanism by which Connexin 43 was regulated had not been described.

Utilizing our stably transduced PAR-1 shRNA cells, we found a significant decrease in Cx-43 expression after PAR-1 silencing as a result of decreased binding of SP-1 and AP-1 to the Connexin 43 promoter [54]. Moreover, we found that silencing PAR-1 results in decreased binding of metastatic melanoma cell lines to human vascular endothelial cells and to human dermal microvascular endothelial cells. Upon re-expressing PAR-1 in PAR-1-silenced cell lines, an increase in attachment was observed. To corroborate that the changes in attachment were due to Connexin 43, we stably silenced Cx-43 in metastatic melanoma cell lines and found decreased attachment of melanoma cells to endothelial cells. This was the first report of PAR-1 regulating Cx-43 expression adding an alternative mechanism to how PAR-1 contributes to the metastatic melanoma phenotype [54].

However, the role of Connexin 43 in melanoma remains controversial. In contrast, to our findings, Cx-43 had previously been found to be decreased in human melanoma cells [62, 63]. These studies focused on the early events involved in melanoma progression in which melanocytes come into contact with keratinocytes via Cx-43 and Cx-26. They further showed that melanoma cells having low Connexin levels were less able to attach to keratinocytes thereby losing their regulation. These studies did not utilize melanoma cells that had developed the capability of metastasizing and in route to the metastatic site. Once melanoma cells are in the circulation, some acquire the capability of arresting and extravasating through the vascular endothelium of the metastatic site. Connexin 43 is crucial for the communication between endothelial cells and tumor cells and plays a role in tumor cell adherence and diapedesis [54, 64].

## PAR-1 REGULATES THE MASPIN TUMOR SUPPRESSOR GENE

One of the other genes identified in our cDNA microarray following PAR-1 silencing was Maspin. Maspin was increased by more than 45-fold as compared to NT shRNA transduced melanoma cells [65]. Maspin is a member of the serine protease inhibitor (serpin) family first isolated from human mammary epithelial cells [66]. Inhibitory serpins bind proteases to its functional domain, the reactive site loop (RSL). The RSL then undergoes cleavage by the protease which causes a conformational change (a stressed to relaxed conformation) in the serine protease inhibitor [67, 68]. The relaxed-state serpin complex inactivates the protease [67].

Maspin was first identified as an inhibitor of tissue-type plasminogen activator [69] and urokinase-type plasminogen activator (uPA) [70, 71]. However, other studies have shown that Maspin does not have a direct serine protease inhibitor function, as its functional domain, the reactive site loop, does not undergo a stressed to relax conformational change essential for protease inhibition by serpins [67, 72]. Recently, Denk et al., proposed that in malignant melanoma, Maspin does function as an inhibitory serpin as it blocks matrix-degrading proteases [73].

Maspin expression has been found to be decreased or lost in several malignancies and is associated with decreased aggressiveness in prostate cancer and breast cancer [74, 75]. Mammary cancer cells that were transfected with Maspin resulted in decreased tumor growth and metastasis in vivo [76].

On the contrary, Maspin has been found to be overexpressed in several cancers, including bladder [77], lung [78], and pancreatic [79] cancers. Furthermore, in ovarian cancer and colon carcinoma, overexpression of Maspin correlates with nuclear translocation [73, 80, 81]. As such, Maspin also seems to have a role as a transcriptional regulator by increasing expression of tumor supportive genes [73, 80]. The nuclear Maspin found in ovarian cancer seems to inhibit the tumor suppressor cytoplasmic functions of Maspin [73, 81]. Thus, the role of Maspin in cancer seems to be tumor type specific and depends on its intracellular localization.

Maspin was recently found to be a tumor suppressor in melanoma. Metastatic melanoma cells were found to have decreased expression levels of Maspin as compared to normal human epidermal melanocytes [73]. Furthermore, this study found that when Maspin was re-expressed in melanoma cells, there was a significant reduction in their invasive capacity.

The mechanism of Maspin regulation, however, has not been described for melanoma. It has been hypothesized that the loss of Maspin in metastatic melanoma tumor specimens might be attributed to an increase in p53, as there is evidence showing overexpression of p53 in metastatic melanoma specimens [82-84]. Furthermore, in several cancers such as breast and prostate cancers, an inverse correlation between p53 and Maspin expression has been suggested [85, 86]. However, Webber and colleagues found no correlation between p53 and Maspin expression when analyzing metastatic melanoma specimens. Thus, the mechanism for Maspin regulation in melanoma has yet to be elucidated [84].

**Figure 2:**
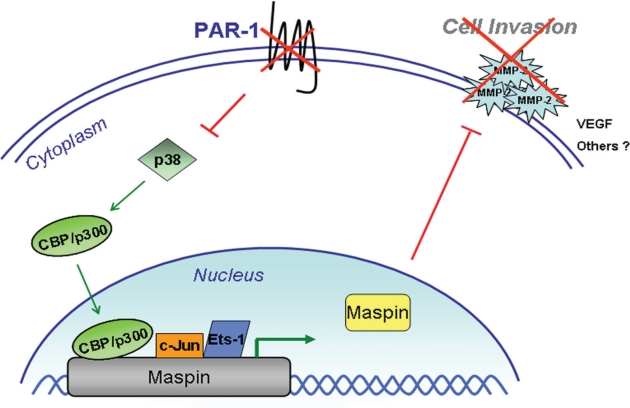
PAR-1 regulates Maspin expression in melanoma Silencing PAR-1 results in decreased activation of p38 MAPK, a known inhibitor of CBP/p300. This results in higher levels of CBP/p300 HAT activity allowing for increased binding of c-Jun and Ets-1 transcription factors to the Maspin promoter. Increased Maspin expression further inhibits cell invasion, through decreased expression and activity of MMP-2, as well as angiogenesis through decreased VEGF expression.

Our laboratory recently published our findings on PAR-1 regulation of the Maspin tumor suppressor gene in melanoma [65]. We found an inverse correlation between PAR-1 and Maspin expression. Highly metastatic melanoma cell lines have high PAR-1 expression and low Maspin levels. Likewise, silencing PAR-1 via lentiviral shRNA, results in increased Maspin expression levels through increased binding of two transcription factors known to regulate Maspin, c-Jun and Ets-1. Interestingly, we did not see a difference in protein expression of c-Jun and Ets-1 after PAR-1 silencing. Rather, we found that silencing PAR-1 inhibits phospho- p38 MAPK which releases the inhibition of the histone acetlytransferase CBP/p300. The increased HAT activity allows for increased binding of Ets-1 and c-Jun to the Maspin promoter and subsequently increased Maspin expression. Our studies further found that the increased in Maspin expression results in a decrease in invasion by inhibiting MMP-2 expression and activity (Figure 2). In vivo studies utilizing Maspin expression vectors showed a significant decrease in melanoma tumor growth and experimental lung metastasis as well as decreased MMP-2, VEGF, and Maspin expression levels from tumor sections. To further corroborate that PAR-1 was regulating Maspin expression in vivo, we stably silenced Maspin in PAR-1-silenced cell lines and showed that levels of melanoma tumor growth and experimental lung metastasis were similar to Non-Targeting shRNA-transduced control cells. Furthermore, levels of Maspin, VEGF and MMP-2 were also increased to levels similar to NT-transduced cells [65].

## CLINICAL IMPLICATIONS

Identifying PAR-1 and its downstream target genes, Connexin 43 and Maspin, as factors involved in melanoma progression is essential, as current therapies for metastatic melanoma are not efficient. The only FDA approved chemotherapeutic agent is Decarbazine even with dismal response rates of 15% [5, 6]. Furthermore, the duration of the response to DTIC is not sustained, often lasting as brief as 5 months [5, 7, 8]. Adjuvant therapies with interferon α or IL-2 for high stage melanoma patients also have limited response rate of less than 20% [9-12]. Our studies suggest that targeting PAR-1 can potentially be used as a target for human melanoma therapy. Furthermore, PAR-1 regulates multiple genes essential for melanoma growth and metastasis such as Connexin 43 which could also potentially serve as novel targets for melanoma therapies. Nevertheless using a single target for therapy has not had success in melanoma. Combining PAR-1 targeted therapy with chemotherapy, anti-angiogenic drugs, pro-apoptotic drugs, or even HDAC inhibitors could potentiate the anti-tumor and anti-metastatic effects seen in our PAR-1 studies. Finding the right combination of therapies to effectively treat metastatic melanoma remains one of the biggest challenges in melanoma research, especially in melanoma cells that do not harbor the BRAF V600E mutation.

Although our studies focus on the effects of PAR-1 on melanoma progression, several other cancers such as breast, prostate, colon and pancreatic cancers have been found to have increased PAR-1 expression with increased progression of disease. As such, targeting PAR-1 in these cancers could also have therapeutic potential.

Taken together our studies show that PAR-1 plays a major role in melanoma progression and that targeting PAR-1 or its downstream target genes could help in treating metastatic melanoma and could have potential benefits in treating several other cancer types as well.
